# Animal foods and mobility limitations in community-dwelling young-old adults: longitudinal analysis of the EpiDoC cohort

**DOI:** 10.1186/s12877-022-03381-0

**Published:** 2022-08-19

**Authors:** Clara Salvador, Ana Maria Rodrigues, Ana Rita Henriques, Maria João Gregório, Helena Canhão, Nuno Mendonça

**Affiliations:** 1 EpiDoC Unit, CEDOC, NOVA Medical School, Universidade Nova de Lisboa, Lisboa, Portugal; 2 Comprehensive Health Research Centre (CHRC), NOVA Medical School, Universidade Nova de Lisboa, Lisboa, Portugal; 3 Direção-Geral da Saúde, Programa Nacional Para a Promoção da Alimentação Saudável, Alameda D. Afonso Henriques 45, 1049-005 Lisboa, Portugal; 4 Faculdade de Ciências da Nutrição e Alimentação da Universidade Do Porto, Porto, Portugal; 5 Population Health Sciences Institute, Newcastle University, Newcastle-upon-Tyne, UK

**Keywords:** Animal foods, Eidos, ADL, Mobility, Function, Protein, Older adults

## Abstract

**Background:**

Nutrition and particularly protein play a role in optimally stimulating muscle protein synthesis and maintaining function. Animal foods are excellent sources of high-quality protein. Therefore, we aimed to determine the association between the consumption of animal foods and mobility limitations in young-old adults.

**Methods:**

The analytic sample was composed of 2860 community-dwelling adults aged 50 and over from a nationally representative longitudinal cohort of Portuguese adults who were followed up to 2.7 years. An animal food intake score was derived from the frequency of consumption of meat, fish, and dairy products. Mobility limitations were defined as the difficulty standing up from a chair, walking, and climbing stairs. To determine the association between animal food intake and mobility limitations mixed effects logistic models were fitted.

**Results:**

Associations between quartiles of animal food intake and mobility limitations (for example, for walking outdoors Quartile 4 v Q1: OR: 0.29; 95%CI: 0.15, 0.56) in unadjusted models were present, but there was no difference in the rate of change of mobility limitations over time in unadjusted models. These associations were no longer present when models were adjusted for sociodemographic, lifestyle and health variables. For example, participants in Q4 of animal food intake were not more or less likely to have difficulty climbing stairs than those in Q1 (OR: 0.95; 95%CI: 0.65, 1.38) nor have a different rate of change over time (OR: 0.86; 95%CI: 0.54, 1.37).

**Conclusions:**

No convincing evidence was found to support an effect of animal foods intake measured at baseline on self-reported mobility limitations over a short period of time.

**Supplementary Information:**

The online version contains supplementary material available at 10.1186/s12877-022-03381-0.

## Background

The ageing process is associated with a gradual and progressive loss of muscle mass, accompanied by a decrease in muscle strength and function which increases the risk for falls, fractures and physical disability [1–3]. The degree of functionality is an important determinant of older people’ quality of life, as it constitutes a strong indicator of independence [4, 5], as well as a strong indicator of disability [6] and, as a consequence, of mortality [7], institutionalization [8] and increased use of health care services [9, 10]. Portugal has seen an increase in life expectancy but Healthy Life Years (HLY), the number of years an individual can expect to live disability-free, has not kept pace. HLY in Portugal has remained constant since 2006 and lower than the European Union 28 average for women and men by 4.0 and 2.8 years, respectively [11]. Nutrition plays an important role in maintaining muscle mass, and adequate intake of protein stands out as an essential nutrient to stimulate muscle protein synthesis [12–14]. Lower protein intake in older adults has been associated with loss of muscle mass and strength, more mobility limitations, loss of independence and mortality in several cohort studies [15–21]. Animal foods are excellent sources of high-quality protein and by not focusing on a single nutrient, the potential cumulative, synergistic, and antagonistic effects on mobility limitations of animal foods may be further explored. However, studies looking at the association between animal foods and mobility limitations, especially in Portugal, are lacking.

## Methods

### Aim of the study

We hypothesized that a higher consumption of animal foods, such as meat, fish, and dairy products, especially due to the high-quality protein content, would lead to a slower decline of muscle strength and functional capacity and eventually translate into fewer mobility limitations (less difficulty with activities of daily living (ADL)). Therefore, we aimed to determine the association between the frequency of animal foods consumption and mobility limitations in Portuguese community-dwelling young-old adults.

### EpiDoC and study population

This study is a longitudinal analysis of the EpiDoC cohort, an observational prospective closed cohort study started in 2011, with a representative sample of the Portuguese adult population (≥ 18 years old) [22, 23]. Population recruitment was conducted by Centro de Estudos e Sondagens de Opinião, Universidade Católica Portuguesa, and multistage random sampling was used for participant selection [22]. The EpiDoC study comprised three waves: EpiDoC 1, where data were collected through face-to-face interviews, and EpiDoC 2 and EpiDoC 3 where data were collected through phone call interviews using a computer-assisted personal interview system [22]. Data from the second and third waves (EpiDoC 2 and EpiDoC 3) were analyzed. EpiDoC 2 (2013–2015), the baseline of this study, enrolled 7591 adults, whereas EpiDoC 3 (2015–2016) was the follow-up wave [22].

The study population was composed by community-dwelling young-old adults (≥ 50 years) living in Portugal (Mainland and the Islands—Madeira and the Azores), from the EpiDoC cohort. Exclusion criteria were age < 50 years (to mimic the Health Retirement Study and its sister studies such as the Survey of Health, Ageing and Retirement in Europe [24] and to include a possible earlier transition to mobility limitations), interview conducted through a proxy or caregiver, missing meat, fish and dairy intake frequency data, and missing mobility limitations (standing up from a chair, walking outdoors and climbing five steps) (Additional file 1). The analytic sample at baseline was 2860–2864 depending on the ADL chosen as outcome, and at follow-up was 1985–1987 with a 30.6% (*n* = 875) attrition (Additional file 1).

### Animal food intake assessment

Dietary intake was assessed at baseline by a non-quantitative food frequency questionnaire that included questions on the frequency of consumption of meat, fish, dairy, fruit, vegetables, and soup. To create the animal food intake score, the frequency of consumption of meat, fish, and dairy products was translated into continuous variables. For meat and fish, the frequencies of 10–14 meals/week, 7–10 meals/week, 4–6 meals/week, 1–3 meals/week, rarely, and never were recoded into 12, 8.5, 5, 2, 0.5, and 0 times/week, respectively. A twice monthly frequency of consumption for the rarely category was assumed, since for meat and fish, the question was related to the number of meals per week instead of the number of times per week. For dairy, the frequencies of everyday, 6 times/week, 3–4 times/week, 1–2 times/week, rarely, and never were recoded into 10.5, 6, 4, 1.5, 0.25, and 0 times/week, respectively. It was assumed that everyday consumption could range from once to twice a day or 7–14 times a week, which results in a middle point of 10.5 times a week. For the rarely frequency, a monthly frequency of consumption was assumed, and it was recoded as 0.25 times per week (once a month). In order to harmonize the frequencies of meat, fish, and dairy consumption, these were transformed into z-scores, and then summed to create an animal food intake score, and transformed into quartiles, where Quartile 1: z-score < -0.45, Q2: -0.45 to 0.01, Q3: 0.02 to 0.37, and Q4: > 0.37 (Additional file 2).

### Mobility limitations

Mobility limitations were assessed through the difficulty with three self-reported ADLs with the Health Assessment Questionnaire (HAQ): standing up from a chair, walking outdoors on flat ground and climbing five steps. The HAQ evaluated difficulty with ADLs over the previous week and ADLs are assessed on a 4-point Likert scale: without any difficulty, with some difficulty, with much difficulty, and unable to do [25]. This instrument also identified specific aids or devices utilized for assistance, as well as help needed from another person or the use of aids [25]. Mobility limitations were operationalized as a binary variable (reporting difficulty or inability to perform these ADLs or no difficulty reported) (Additional file 2).

### Other variables

Sociodemographic variables, including age, sex, nomenclature of territorial units for statistics II (NUTS II) region (North, Centre, Lisbon, Alentejo or Algarve, and Azores or Madeira) and years of education were collected [22]. Smoking habits (never smoked, former and current smoker), alcohol intake (alcohol drinkers or not) and frequency of physical exercise (low, medium, high) were also collected [22]. Self-reported weight and height were used to calculate the body mass index (BMI) (weight in kg/height in m^2^). The number of chronic diseases was calculated by summing the presence or absence of diabetes, respiratory, cardiac, neurological, mental, oncological, and rheumatic diseases [22]. Data was also collected on hospitalizations events in the previous 12 months since last contact [22]. Dietary intake variables included the number of meals per day, and the frequency of vegetables and fruit per week [22]. Details about the operationalization of all used variables are in Additional file 2.

### Statistical analyses

Data cleaning and quality control were performed before analysis. Normality was assessed by Q-Q plots: normally and non-normally distributed variables are presented as means and SDs, and medians and interquartile ranges (IQR), respectively, and categorical data as percentages and frequency. Non-difference between missing and non-missing variables according to the analytic sample were assessed with chi-squared test (χ2) for categorical variables and t-test/ Mann–Whitney for continuous variables along with the effect size and, SD or IQR. Non-difference between quartiles of animal food intake were assessed in the same manner but ANOVA/ Kruskal–Wallis for continuous variables.

To determine the association between animal foods and mobility limitations (difficulty or inability to stand up from a straight chair, to walk outdoors on flat ground or climb five steps), we fitted mixed effects logistic models using adaptive Gaussian quadrature with the *GLMMadaptive* package (v 0.8.0) [26] in R v3.6.2. The mixed effects logistic models used two observations from the same participant at different time-points. These mobility limitations are prevalences as models with incidence did not converge. Model l included id, time since the start of the study (random effects), quartiles of animal foods intake and its interaction with time (fixed-effects); Model 2 was adjusted for the previous variables plus age, sex, NUTS II and education (fixed-effects); Model 3 was further adjusted for physical activity, number of meals, BMI, and vegetables and fruit consumption (fixed-effects); and Model 4 was also adjusted for number of chronic diseases and recent hospitalizations (fixed-effects). Quartile 1 of animal foods intake was used as reference for all models. Time, age, BMI, and number of chronic diseases were centered prior to analyses. The random effects (id and time) were considered uncorrelated to reduce the complexity of the models and achieve convergence.

For sensitivity analyses the final model was further adjusted for the use of helping devices, or smoking, or alcohol intake, or imputing missing BMI values, or excluding participants who reported having neurological disease or those who had been hospitalized in the previous visit or those who were younger than 65 years old, or using frequency of meat, fish, and dairy intake separately instead of the animal foods score. Point estimates and confidence intervals were used to assess statistical and clinical significance. Results are presented as odd ratios (OR) and 95% confidence intervals (CI).

## Results

### Missing and non-missing dietary intake and mobility limitations

At baseline, more women (70.2% v 50.9%), more participants who had never smoked (82.3% v 60.3%), who did not drink alcohol (29.2% v 60.8%), who had lower physical activity (78.7% v 60.8%), and who had more mobility limitations (e.g., walking outdoors: 52.6% v 31.5%) had missing dietary intake data or missing mobility limitations or relied on a caregiver to answer. Those who had missing data (*n* = 3109) and those who did not (*n* = 2860) were also statistically different for levels of education, BMI, recent hospitalization, number of chronic diseases and use of helping devices but arguably not clinically meaningful. Age was similar between those excluded and included in the analytic sample (*n* = 2860) (Additional file 3).

### Animal foods, sociodemographic and health characteristics

The analytic sample consisted of 2860 men and women with a median age of 66.6 years (IQR: 59.2, 74.7) at baseline (Table 1). The maximum follow-up time was 2.7 years (mean: 1.2, SD: 0.3 years). Participants with a lower intake of animal foods also had lower frequencies of meat, fish, and dairy intake. For example, 7.8%, 4.2% and 44.3% of those in Q1 of animal foods intake ate meat ≥ 7 times/ week, fish ≥ 7 times/ week, and dairy ≥ 6 times/ week, respectively, while 74.6%, 62.9% and 99.6% of those in Q4 ate meat ≥ 7 times/ week, fish ≥ 7 times/ week and dairy ≥ 6 times/ week, respectively (Table 1).

**Table 1:** Baseline sociodemographic and health characteristics of participants by quartile of animal and animal-derived food intake

	**All** (*n* = 2860)	Missing (%)	**Q1** (*n* = 742)	**Q2** (*n* = 681)	**Q3** (*n* = 728)	**Q4** (*n* = 709)	*p*
Sociodemographic							
Age, y, median (IQR)	66.6 (59.2, 74.7)	0.0	69.7 (61.9, 77.5)	67.2 (59.2, 75.1)	66.2 (60.1, 73.9)	64.0 (56.9, 71.2)	< 0.001
Women, % (n)	50.9 (1455)	0.0	55.5 (412)	53.6 (365)	49.5 (360)	44.9 (318)	< 0.001
NUTS II		0.0					< 0.001
North	32.4 (927)		32.9 (244)	33.3 (227)	31.0 (226)	32.4 (230)	
Centre	25.3 (724)		24.0 (178)	23.3 (159)	30.4 (221)	23.4 (166)	
Lisbon	19.1 (545)		15.9 (118)	24.2 (165)	19.0 (138)	17.5 (124)	
Alentejo	5.7 (164)		5.3 (39)	5.0 (34)	4.8 (35)	7.9 (56)	
Algarve	3.0 (86)		2.7 (20)	2.9 (20)	1.8 (13)	4.7 (33)	
Azores	5.4 (154)		5.1 (38)	4.7 (32)	4.7 (34)	7.1 (50)	
Madeira	9.1 (260)		14.2 (105)	6.5 (44)	8.4 (61)	7.1 (50)	
Education, years, % (n)		0.3					< 0.001
≤ 9	81.4 (2320)		87.4 (646)	80.5 (546)	80.2 (582)	77.2 (546)	
10–12	9.9 (281)		6.2 (46)	11.5 (78)	10.2 (74)	11.7 (83)	
≥ 13	8.7 (249)		6.4 (47)	8.0 (54)	9.6 (70)	11.0 (78)	
Lifestyle							
BMI, kg/m^2^, median (IQR)	26.8 (24.3, 29.5)	6.5	26.7 (24.1, 29.4)	27.1 (24.7, 30.0)	26.5 (24.4, 29.4)	26.8 (24.3, 29.4)	0.115
Smoker, % (n)		0.1					0.002
No	60.3 (1723)		64.8 (481)	62.7 (427)	58.3 (423)	55.3 (392)	
Former	28.7 (821)		26.1 (194)	28.0 (191)	30.3 (220)	30.5 (216)	
Current	11.0 (314)		9.0 (67)	9.3 (63)	11.4 (83)	14.2 (101)	
Alcohol drinker, % (n)	60.8 (1734)	0.2	55.5 (411)	58.7 (399)	63.9 (463)	65.1 (461)	< 0.001
Physical exercise, % (n)		0.6					< 0.001
Lower	60.8 (1728)		68.6 (503)	60.9 (412)	59.3 (430)	54.1 (383)	
Medium	10.4 (295)		7.5 (55)	10.6 (72)	11.4 (83)	12.0 (85)	
Higher	28.8 (820)		23.9 (175)	28.5 (193)	29.2 (212)	33.9 (240)	
Health							
Chronic diseases, mean (SD)	1.2 (1.1)	0.0	1.3 (1.2)	1.2 (1.2)	1.2 (1.1)	1.0 (1.1)	0.001
Hospitalized recently, % (n)	20.7 (590)	0.6	22.3 (164)	20.1 (137)	20.9 (151)	19.6 (138)	0.636
Mobility limitations, % (n)							
Baseline							
Standing up from chair, % (n)	33.9 (970)	0.0	35.8 (266)	37.0 (252)	31.5 (229)	31.5 (223)	0.047
Walking outdoors, % (n)	31.5 (900)	0.0	34.8 (258)	33.0 (225)	29.4 (214)	28.6 (203)	0.035
Climbing steps, % (n)	38.0 (1088)	0.0	43.7 (324)	38.9 (265)	36.8 (268)	32.6 (231)	< 0.001
Uses helping devices, % (n)	10.1 (289)	0.3	15.7 (116)	8.2 (56)	9.5 (69)	6.8 (48)	< 0.001
Follow-up							
Standing up from chair, % (n)	30.6 (607)	0.1	36.3 (185)	31.2 (148)	28.3 (143)	26.4 (131)	0.004
Walking outdoors, % (n)	29.8 (592)	0.0	37.5 (191)	29.1 (138)	27.1 (137)	25.4 (126)	< 0.001
Climbing steps, % (n)	34.7 (690)	0.0	42.0 (214)	34.9 (166)	33.2 (168)	28.6 (142)	< 0.001
Uses helping devices, % (n)	9.4 (186)	0.4	15.0 (76)	8.4 (40)	7.3 (37)	6.7 (33)	< 0.001
Incidence of mobility limitations, % (n)							
Standing up from chair, % (n)	14.2 (284)	30.2	12.9 (67)	14.3 (68)	15.7 (80)	13.9 (69)	0.633
Walking outdoors, % (n)	10.5 (210)	30.1	10.4 (54)	9.7 (46)	10.8 (55)	11.1 (55)	0.905
Climbing steps, % (n)	10.9 (219)	30.1	10.6 (55)	10.7 (51)	12.9 (66)	9.5 (47)	0.348
Dietary Intake							
Number of meals per day, % (n)		0.3					< 0.001
2	5.9 (169)		9.1 (67)	7.1 (48)	3.3 (24)	4.2 (30)	
3	44.8 (1276)		51.0 (377)	44.5 (302)	43.2 (314)	40.0 (283)	
4	31.6 (901)		26.5 (196)	30.8 (209)	35.9 (261)	33.2 (235)	
≥ 5	17.7 (505)		13.4 (99)	17.6 (119)	17.6 (128)	22.5 (159)	
Vegetables, times/week, % (n)		0.1					< 0.001
Rarely/ Never	3.4 (98)		5.3 (39)	3.8 (26)	2.5 (18)	2.1 (15)	
1–2	7.4 (212)		11.1 (82)	8.5 (58)	5.4 (39)	4.7 (33)	
3–5	22.5 (643)		27.5 (204)	21.7 (148)	20.9 (152)	19.6 (139)	
≥ 6	66.7 (1905)		56.2 (417)	65.9 (449)	71.3 (518)	73.6 (521)	
Fruit, times/week, % (n)		0.1					< 0.001
Rarely/ Never	2.9 (84)		5.3 (39)	3.1 (21)	2.2 (16)	1.1 (8)	
1–2	3.7 (106)		5.4 (40)	3.5 (24)	3.2 (23)	2.7 (19)	
3–5	8.5 (242)		10.6 (79)	8.7 (59)	7.0 (51)	7.5 (53)	
≥ 6	84.9 (2426)		78.7 (584)	84.7 (575)	87.6 (638)	88.7 (629)	
Meat, times/week, % (n)		0.0					< 0.001
Rarely/ Never	3.6 (102)		7.7 (57)	3.2 (22)	2.1 (15)	1.1 (8)	
1–3	32.0 (915)		65.0 (482)	38.9 (265)	13.6 (99)	9.7 (69)	
4–6	32.9 (941)		19.5 (145)	43.0 (293)	54.9 (400)	14.5 (103)	
≥ 7	31.5 (902)		7.8 (58)	14.8 (101)	29.4 (214)	74.6 (529)	
Fish, times/week, % (n)		0.0					< 0.001
Rarely/ Never	3.4 (97)		8.1 (60)	2.2 (15)	1.8 (13)	1.3 (9)	
1–3	36.8 (1053)		67.9 (504)	40.8 (278)	18.0 (131)	19.7 (140)	
4–6	34.9 (999)		19.8 (147)	50.2 (342)	54.4 (396)	16.1 (114)	
≥ 7	24.9 (711)		4.2 (31)	6.8 (46)	25.8 (188)	62.9 (446)	
Dairy, times/week, % (n)		0.0					< 0.001
Rarely/ Never	9.8 (279)		27.8 (206)	6.2 (42)	4.3 (31)	0.0 (0)	
1–2	5.6 (159)		15.4 (114)	4.4 (30)	2.1 (15)	0.0 (0)	
3–5	8.7 (248)		12.5 (93)	16.7 (114)	5.2 (38)	0.4 (3)	
≥ 6	76.0 (2174)		44.3 (329)	72.7 (495)	88.5 (644)	99.6 (706)	

Mobility limitations refer to difficulty or inability standing up from a straight chair, walking outdoors on flat ground and climbing five steps. Incidence of mobility limitations refers to those who transitioned from not having mobility limitations at baseline to having mobility limitations at follow-up. At baseline, Q1: z-score < -0.45, Q2: -0.45 to 0.01, Q3 0.02 to 0.37, and Q4: > 0.37. Non-difference between quartiles was assessed with chi-squared test (χ2) for categorical variables and ANOVA/ Kruskal–Wallis for continuous variables along with the effect size and, SD or IQR. NUTS II is presented as seven regions, but Alentejo and Algarve were collapsed into one region, as well as Azores and Madeira for the model building process

*BMI* Body mass index, *IQR* Interquartile range, *NUTS II* Nomenclature of Territorial Units for Statistics II, *Q* Quartile, *SD* Standard deviation, *y* Years

At baseline, participants who had lower intake of animal foods were older, were more likely to be women (e.g. Q1: 55.5% v Q4: 44.9%), had slightly lower levels of education (e.g. Q1: 87.4% v Q4: 77.2% with ≤ 9 years of education), more had never smoked (e.g. Q1: 64.8% v Q4: 55.3%), less were alcohol drinkers (e.g. Q1: 55.5% v Q4: 65.1%), had lower levels of physical exercise (e.g. Q1: 68.6% v Q4: 54.1%), had slightly less number of meals per day and had less fruit and vegetable intake per week than those with higher intake of animal foods (Table 1). NUTS II and the number of chronic diseases were statistically different between quartiles but arguably not clinically meaningful. BMI and recent hospitalization were similar between quartiles. The percentage of mobility limitations remained similar or decreased between the baseline and follow-up wave (e.g., walking outdoors in Q3 of animal food intake declined from 33.0% at baseline to 29.1% at follow-up) (Table 1).

### Animal foods and mobility limitations

**Fig. 1:**
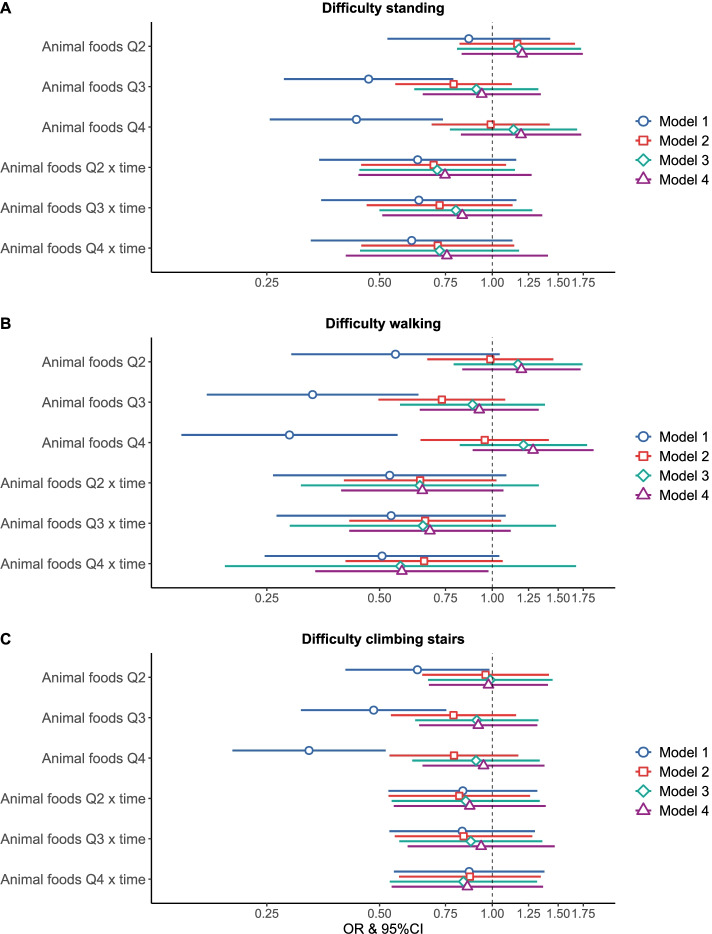
Odd ratios and 95% confidence intervals for the odds of having a mobility limitation over time according to quartiles of the consumption of animal foods. The figure shows the odds (and 95%CI) of having a mobility limitation (standing up from a chair (**A**), walking outdoors (**B**) and climbing five stairs (**C**)) according to quartiles of animal food intake and the interaction with time. The analytic sample consisted of 2860 participants at baseline. Model 1 is adjusted for time, animal foods intake and an interaction between both; Model 2 is further adjusted for age, sex, NUTS II, and education; Model 3 is further adjusted for physical activity, number of meals, BMI, and vegetables and fruit consumption; and Model 4 is also adjusted for number of chronic diseases and recent hospitalizations. Q1 was used as reference for all models

We found an association between quartiles of animal food intake (Q1 referent) and mobility limitations (standing up from a chair, walking outdoors and climbing steps) (for example, for walking outdoors Q4 v Q1: OR: 0.29; 95%CI: 0.15, 0.56), but no difference in the rate of change of mobility limitations (quartiles of animal foods x time) in unadjusted models (model 1) (Fig. 1). There was no longer an association when more complex models adjusted for age, sex, NUTS II, education (model 2), physical activity, number of meals, BMI, vegetable, and fruit intake (model 3), and number of chronic diseases and recent hospitalizations (model 4) were fitted. For example, participants in Q4 of animal food intake were not more or less likely to have difficulty climbing stairs than those in Q1 (OR: 0.95; 95%CI: 0.65, 1.38) nor have a different slope over a maximum of 2.7 years (OR: 0.86; 95%CI: 0.54, 1.37). Participants with higher animal foods intake may be less likely to have difficulty walking outdoors over time, but the associations were non-statistically significant or only slightly (for example, Q4 x time v Q1 x time; OR: 0.57; 95%CI: 0.34–0.98) (Fig. 1).

### Sensitivity analysis

Final models were further adjusted for the use of helping devices, or smoking, or alcohol intake, or imputing missing BMI values, or excluding participants who reported having a neurological disease or those who had been hospitalized in the previous visit or those who were younger than 65 years old, or using frequency of meat, fish, and dairy intake separately instead of the animal foods score but the results did not change significantly.

## Discussion

In this study, no associations were found between higher consumption of animal foods at baseline and mobility limitations (standing up from a chair, walking outdoors or climbing steps) and in the rate of decline of mobility limitations over a maximum of 2.7 years. More complex models and sensitivity analysis confirmed the findings.

Bradlee *et al.* found that higher intake of animal-protein foods (red meats, poultry, fish, and dairy), alone but especially in combination with a physically active lifestyle, were associated with preservation of muscle mass and functional performance in middle-aged adults (median of 52 and 55 years for muscle mass and functional performance, respectively) [27]. As opposed to animal foods, beneficial effects of plant-based protein foods were only evident in physically active adults [27]. As compared to our study, the latter had a much longer follow-up period and a more complete food frequency questionnaire, which may provide reasons why no similar results were observed.

Increased risk of colorectal cancer, type 2 diabetes, cardiovascular disease, and all-cause mortality was found in those that had a high consumption of red and processed meat [28–30]. In turn, fish consumption was associated with a reduced risk of all-cause mortality [31] and of cardiovascular disease events [32]. Moreover, daily intake of low or non-fat dairy may be associated with higher bone mineral density and with fewer fractures in older adults [33]. In our study, the animal food intake score was created from the frequencies of meat, fish, and dairy intake. Adverse effects of red and processed meat, as well as beneficial effects of fish and dairy may influence functional outcomes in older adults and bias our results towards the null. All models were re-fitted to include a term for the frequency of meat, fish, and dairy intake as exposure separately instead of the animal foods score, but conclusions were similar.

Animal foods are good sources of high-quality protein, and we initially hypothesized that a possible beneficial effect of animal foods on mobility limitations would be in great part due to the higher protein content. Several studies have found that higher protein consumption was associated with better functionality in older adults [20, 21, 34–43] while only a few others have not [35, 44]. For example, in the Health ABC study, lower protein intake was associated with increased risk of mobility limitation in community-dwelling, initially well-functioning older adults [36]. Compared to participants in the upper tertile of protein intake (≥ 1.0 g/kg body weight/d), participants in the lower two tertiles of protein intake (< 0.7 and 0.7—< 1.0 g/kg body weight/d) were at greater risk of developing a mobility limitation over 6 years of follow-up (RR (95% CI): 1.86 (1.41–2.44) and 1.49 (1.20–1.84), respectively) [36]. Having the distribution of animal, plant, and total protein intake between quartiles of animal food intake might have explained some of the general null findings.

The nationally representative population, the large sample size and the robust study design are all major strengths of this study. Additionally, confounders were collected prospectively and, whenever possible, used as time-dependent variables. The analysis also included three outcomes (standing up from a chair, walking outdoors or climbing steps), and extensive sensitivity analyses were performed.

There are weaknesses that should be considered when drawing conclusions from our study. At baseline and follow-up, data were collected by telephone interviews, and self-reported data, especially dietary intake data, may have been partially misreported. However, protein-rich foods, such as animal foods, are not commonly underreported, unlike snacks or sweets, which means that differential misclassification of our exposure is less likely compared to other foods [45]. The food frequency questionnaire did not include the frequency of consumption of all foods, therefore, inclusion of eggs and egg products, and estimating nutritional intake, namely protein intake and total energy intake was not possible. Inclusion of the number of meals per day was added as a proxy for total food consumption/energy intake. Additionally, total meat/poultry/fish intake tend to be overestimated in national dietary surveys, when disaggregation of mixed dishes is not taken into account, and the same might have happened in this study [46]. Food frequency questionnaire was only available at baseline (2013–2015), and we had to assume that the frequency of consumption of animal foods was constant or changed equally until follow-up. The follow-up wave (2015–2016) was on average 1.2 years after baseline, so this assumption seems more plausible than if it was a larger gap. However, this is also a limitation as we may not have captured the incidence of mobility limitations in a young-old population with median age of 67 (59 to 75) years. Complete mortality data was unavailable in EpiDoC, and it is possible that those lost at follow-up were not missing at random. In fact, the percentage of participants with mobility limitations remained similar or decreased between baseline and follow-up. This may be due to self-reported bias and reflect other transitions (e.g., recovery) or that those who were worse dropped from the study. Although attempts were made to avoid reverse-causality, lower consumption of animal foods may be a consequence of mobility limitations. Limitations in our study, especially the short follow-up duration and self-reported nature of our exposure and outcome, as well as the other variables, may present possible explanations for the general null findings. Animal protein intake was assessed using a Food Frequency Questionnaire that has not been validated but based on the expert advice of a group of nutritionists, which may present itself as a considerable limitation of this study. Furthermore, our analysis required participants to have dietary intake data and by excluding those without the sample of ≥ 50 years old was reduced by half. The smaller number of participants included in the analysis due to lack of dietary assessment may have reduced the generalization of the findings.

## Conclusions

No convincing evidence was found to support an effect of animal foods intake on self-reported mobility limitations in this study. Further studies with longer follow-up and more detailed exposures are needed to confirm our results in this setting.

## Supplementary Information

**Additional file 1. **Flowchart of EpiDoC and the exclusion criteria for the analytic sample.

**Additional file 2. **Description and operationalization of selected variables used in the models.

**Additional file 3. **Baseline sociodemographic and health characteristics of participants missing dietary intake or mobility limitations, and participants included in the analytic sample.

## Data Availability

The codebook and analytic code are available pending request from the authors while the dataset is available pending application and approval by the EpiDoC Coordinator—Ana Maria Rodrigues (ana.m.rodrigues@nms.unl.pt).

## Abbreviations

ADL: Activities of daily living
BMI: Body mass index
CI: Confidence interval
IQR: Interquartile range
NUTS II: Nomenclature of territorial units for statistics II
OR: Odds ratio
Q: Quartile
ref: Reference group
SD: Standard deviation
