# Supplementary material for: Animal foods and mobility limitations in community-dwelling young-old adults: longitudinal analysis of the EpiDoC cohort
Source: BMC Geriatr. 2022 Aug 19;22:687. doi: 10.1186/s12877-022-03381-0 (PMC9389693; doi:10.1186/s12877-022-03381-0)
Supplement: Supplementary file 1 — Additional file 1. Flowchart of EpiDoC and the exclusion criteria for the analytic sample. [file 12877_2022_3381_MOESM1_ESM.pdf]

Additional file 1. Flowchart of EpiDoC and the exclusion criteria for the analytic sample.

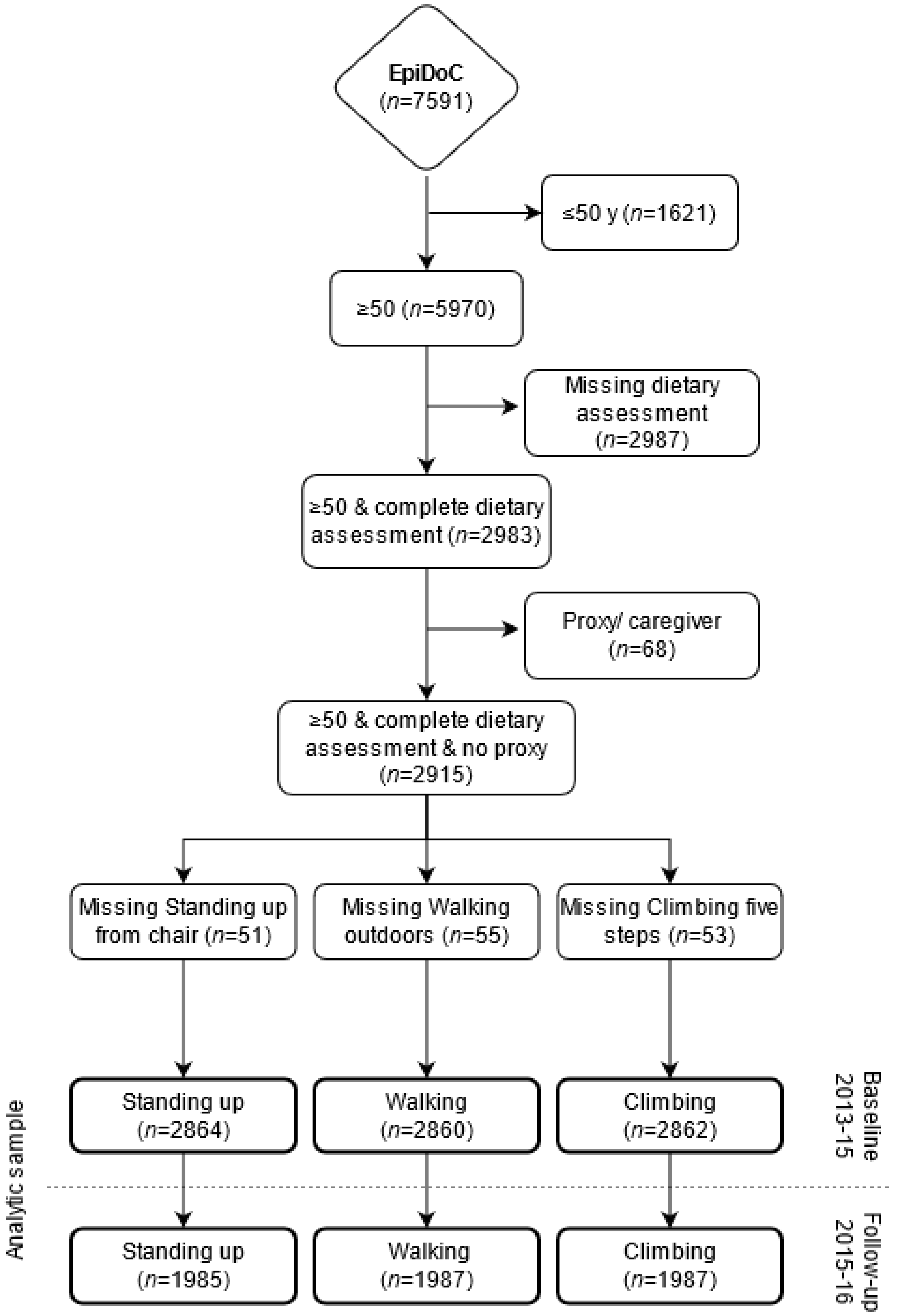

Standing up, walking, and climbing steps refer to the difficulty or inability to stand up from a straight chair, to walk outdoors on flat ground and climb five steps, respectively. Availability of data is specific to this study's hypothesis and analytic strategy and not the whole EpiDoC cohort. y, years.
