# Supplementary material for: Animal foods and mobility limitations in community-dwelling young-old adults: longitudinal analysis of the EpiDoC cohort
Source: BMC Geriatr. 2022 Aug 19;22:687. doi: 10.1186/s12877-022-03381-0 (PMC9389693; doi:10.1186/s12877-022-03381-0)
Supplement: Supplementary file 2 — Additional file 2. Description and operationalization of selected variables used in the models. [file 12877_2022_3381_MOESM2_ESM.pdf]

Additional file 2. Description and operationalization of selected variables used in the models.

Variable	Description	Wave	Coding
id	(Pseudo-)anonymous unique identifier	Baseline, follow-up	string
		**Exposure**	
Animal foods	Quartiles of the self-reported consumption of animal foods (meat, fish, and dairy)	Baseline	0= Quartile 1<br>1= Quartile 2<br>2= Quartile 3<br>3= Quartile 4
		**Outcome**	
Standing up from chair	Self-reported difficulty to stand up from a straight chair	Baseline, follow-up	0= without any difficulty<br>1= unable/with difficulty
Walking outdoors	Self-reported difficulty to walk outdoors on flat ground	Baseline, follow-up	0= without any difficulty<br>1= unable/with difficulty
Climbing steps	Self-reported difficulty to climb five steps	Baseline, follow-up	0= without any difficulty<br>1= unable/with difficulty
		**Possible Confounders**	
Time	Time since baseline	Baseline, follow-up	numerical
Age	Age in years since date of birth	Baseline, follow-up	numerical
Sex	Biological sex	Baseline	0= man<br>1= woman
NUTS II	Nomenclature of Territorial Units for Statistics II	Baseline	1= North<br>2= Center<br>3= Lisbon<br>4= Alentejo or Algarve<br>5= Azores or Madeira
Education	Total number of years of full-time education	Baseline	0= ≤9 years<br>1= 10-12 years<br>2= ≥13 years
Body mass index	Self-reported body weight (kg)/ height (m)^2	Baseline, follow-up	numerical
Smokers	Smoking habits	Baseline	0= never smoked<br>1= former smoker<br>2= current smoker
Alcohol intake	Alcohol habits	Baseline	0= non-drinker

Variable	Description	Wave	Coding
			1= drinker
Physical activity	Tertiles of the self-reported frequency of physical activity	Baseline, follow-up	0= no physical activity/ sporadically/occasionally<br>1= 1-2 times/week<br>2= ≥3 times/week or everyday
Meals	Number of meals per day	Baseline	0= 2 meals<br>1= 3 meals<br>2= 4 meals<br>3= ≥5 meals
Vegetables	Consumption of vegetables per week	Baseline	0= rarely or never<br>1= 1-2 times/week<br>2= 3-5 times/week<br>3= every day or 6 times/week
Fruit	Fruit consumption per week	Baseline	0= rarely or never<br>1= 1-2 times/week<br>2= 3-5 times/week<br>3= every day or 6 times/week
Chronic diseases	Number of self-reported chronic diseases[1]	Baseline, follow-up	numerical
Hospitalized recently	Hospitalized since last contact	Baseline, follow-up	0= no<br>1= yes
Helping devices	Self-reported use of special helping devices such as canes, walkers, wheelchairs, and others	Baseline, follow-up	0= does not use<br>1= uses

Availability of data is specific to this study's hypothesis and analytic strategy and not the whole EpiDoC cohort. [1] Includes the presence or absence of diabetes, respiratory, cardiac, neurological, mental, oncological, and rheumatic diseases.
