# Supplementary material for: Animal foods and mobility limitations in community-dwelling young-old adults: longitudinal analysis of the EpiDoC cohort
Source: BMC Geriatr. 2022 Aug 19;22:687. doi: 10.1186/s12877-022-03381-0 (PMC9389693; doi:10.1186/s12877-022-03381-0)
Supplement: Supplementary file 3 — Additional file 3. Baseline sociodemographic and health characteristics of participants missing dietary intake or mobility limitations, and participants included in the analytic sample. [file 12877_2022_3381_MOESM3_ESM.pdf]

Additional file 3. Baseline sociodemographic and health characteristics of participants missing dietary intake or mobility limitations, and participants included in the analytic sample.

	Missing (*n*=3109)	Analytic sample (n=2860)	*p*
Sociodemographic			
Age, y, median (IQR)	66.5 (58.8, 74.4)	66.6 (59.2, 74.7)	0.234
Women, % (n)	74.2 (2307)	50.9 (1455)	<0.001
NUTS II			<0.001
North	25.6 (797)	32.4 (927)	
Centre	16.2 (504)	25.3 (724)	
Lisbon	22.9 (712)	19.1 (545)	
Alentejo	8.9 (278)	5.7 (164)	
Algarve	4.3 (133)	3.0 (86)	
Azores	11.2 (349)	5.4 (154)	
Madeira	10.8 (336)	9.1 (260)	
Education, years, % (n)			<0.001
≤ 9	86.3 (2669)	81.4 (2320)	
10-12	7.5 (231)	9.9 (281)	
≥ 13	6.2 (193)	8.7 (249)	
Lifestyle			
BMI, kg/m^2, median (IQR)	27.4 (24.7, 30.9)	26.8 (24.3, 29.5)	<0.001
Smoker, % (n)			<0.001
No	82.3 (1109)	60.3 (1723)	
Former	11.0 (148)	28.7 (821)	
Current	6.8 (91)	11.0 (314)	
Alcohol drinker, % (n)	29.2 (392)	60.8 (1734)	<0.001
Physical exercise, % (n)			<0.001
Lower	78.7 (891)	60.8 (1728)	
Medium	7.4 (84)	10.4 (295)	
Higher	13.9 (157)	28.8 (820)	
Health			
Chronic diseases, mean (SD)	0.7 (1.1)	1.2 (1.1)	<0.001
Hospitalized recently, % (n)	15.9 (225)	20.7 (590)	<0.001
Mobility limitations % (n)			
Standing up from chair	60.2 (739)	33.9 (970)	<0.001
Walking outdoors	52.6 (643)	31.5 (900)	<0.001
Climbing steps	52.7 (645)	38.0 (1088)	<0.001
Uses helping devices	13.4 (164)	10.1 (289)	0.003

Difficulty/ unable to walk outdoors on flat ground was used to define missing mobility limitations since it was missing for more participants (n=1886) than the other mobility limitations. Most participants were not included in the analytic sample (n=2860) because of missing dietary intake (n=2987 *v* n=1886 missing mobility limitations), a few were also caregivers (n=68) and a few also had missing mobility limitations (n=55). Non-difference between missing and non-missing was assessed with chi-squared test

(χ2) for categorical variables and t-test/ Mann-Whitney for continuous variables along with the effect size and, SD or IQR. BMI, body mass index; IQR, interquartile range; NUTS II, Nomenclature of Territorial Units for Statistics II; Q, quartile; SD, standard deviation; y, years.
